# Prevalence of Alexithymia in Patients with Medically Unexplained Physical Symptoms: A Cross-sectional Study in Egypt

**DOI:** 10.2174/1745017902117010136

**Published:** 2021-10-15

**Authors:** Ahmed Rady, Roa Gamal Alamrawy, Ismail Ramadan, Mervat Abd El Raouf

**Affiliations:** 1 Department of Psychiatry, Alexandria University, School of Medicine, Alexandria, Egypt; 2 Mamoura Psychiatric Hospital, Secretariat of Mental Health and Addiction Treatment, Alexandria, Egypt; 3 Department of Neurology, Alexandria University School of Medicine, Alexandria, Egypt

**Keywords:** Medically unexplained physical symptoms, Alexithymia, Somatic symptoms, Toronto alexithymia scale (TAS-20), Patient health questionnaire (PHQ-15), Dyspepsia

## Abstract

**Background:**

There is a high incidence of alexithymia in people who report medically unexplained symptoms. There have been limited studies on the prevalence of alexithymia in patients with medically unexplained physical symptoms (MUPS) in various ethnic and cultural backgrounds.

**Objective:**

This study aimed to estimate the prevalence of alexithymia in patients with MUPS and examine their socio-demographic data.

**Methods:**

In this cross-sectional study, 196 patients with MUPS were recruited from tertiary care internal medicine and neuropsychiatry clinics during the first quarter of 2019. Patients completed a structured interview; socio-demographic and medical history data were collected. Somatic symptom severity was assessed using the Arabic version of the Patient Health Questionnaire (PHQ-15). Alexithymia was assessed using the Arabic version of the Toronto Alexithymia Scale.

**Results:**

General fatigue was the most common complaint observed, followed by headache and dyspepsia. In addition, 73.5% of patients had a high Patient Health Questionnaire score, 17.9% had somatic symptoms of medium severity, while 8% and 0.5% had low and marginal somatic symptoms, respectively. Alexithymia was presented in 49.5%, 22.9% had no alexithymia, and 27.6% had borderline/intermediate alexithymia.A weak positive correlation (r<0.4) was found between somatic symptom severity and alexithymic psychopathology (r=0.277;p<0.05). Only the ‘difficulty identifying feelings’ dimension of alexithymic psychopathology was positively correlated with the severity of somatic symptoms (r=0.271;p<0.05).

**Conclusion:**

Alexithymia is associated with the development of MUPS.

## 1 INTRODUCTION

In general practice, it is normal to encounter patients with no medical explanation for their symptoms and no medical diagnosis. According to the literature, incidences of this phenomenon range from 10 to 50%, with significantly higher incidences in specialty clinical settings [1-3]. A cross-sectional design has most often been employed in investigations involving patients with medically unexplained physical symptoms (MUPS). These investigations aim to explore whether symptoms could be signs of a psychiatric illness or linked to common predisposing or triggering factors for psychiatric illness. Medically unknown clinical manifestations are often linked with psychological pathology in patient groups. Indeed, anxiety, depression, and dissociative symptoms have been shown to be linked with MUPS [4]. This is of clinical significance because most patients with MUPS are able to comprehend that their physical symptoms may be associated with stress or anxiety [5].

MUPS are also strongly associated with personality pathology; numerous studies have reported that personality defects are often associated with somatoform disorders [6]. In addition, it has been observed that MUPS are correlated with personality traits such as hypochondriac concern and excessive disease conduct [7]. Personality pathology is assumed to be a predisposing factor in the precipitation of MUPS, and it is thought that alexithymia plays an essential part in it [8].

Alexithymia has been described as a personality trait characterized by difficulty in distinguishing and understanding emotions, a reduced capacity for creativity and imagination, and externally focused thought rather than focusing on the experience of reality [9]. Given that patients with alexithymia are not expected to fully understand that physical symptoms can be somatic representations of emotions, they are considered to be more likely to falsely attribute physical signs to physical illness and to request medical attention for symptoms with no underlying medical cause. Therefore, alexithymia is known to be a predisposing and persistent factor in the occurrence of MUPS, contributing to one of the somatoform disorders [10, 11]. Various studies, usually non-clinical trials, have reported a correlation between alexithymia and multiple somatization interventions. Not all patients with MUPS have alexithymic features of the same magnitude; thus, the prognosis of patients with severe alexithymia is considered to be less positive than that of patients who have milder alexithymia [12-14].

Given the limited number of studies on the contribution of alexithymia in the persistence of MUPS, the present study investigated the prevalence of alexithymia in patients with unexplained physical symptoms. Our study was conducted in Egypt, where there is a lack of epidemiological studies; notably, alexithymia is also a culture-sensitive issue and can vary according to cultural differences [15].

## 2 MATERIALS AND METHODS

### 2.1 Participants

This was a cross-sectional study conducted on 196 patients with MUPS. Patients were recruited from tertiary care internal medicine and neuropsychiatry clinics during the first quarter of 2019. Ethical approval was obtained from the research review board at Alexandria University’s School of Medicine. All participants gave written consent.

The inclusion criteria for participation in this study are as follows: Patients presented with persistent MUPS for which no identifiable medical cause was found after proper medical examination and investigations, they had experienced MUPS for at least for 3 months, and these had led to dysfunction, and were adults aged less than 60 years.

The exclusion criteria are as follows: Pregnant women, patients with immune compromising diseases, those with active substance abuse (urine screening for cannabis, opiates, amphetamine, cocaine, barbiturate, benzodiazepines), patients with psychotic symptoms (assessed by consultant psychiatrists), and patients in whom the current medical condition fully contributed to the presenting symptoms.

All patients provided signed informed consent. Details about the enrolled patients were collected prior to the start of the study. The selected patients were subjected to a structured interview.

### 2.2 Demographic Data and Medical History

#### 2.2.1 Socio-demographic Data

Data on age, sex, residence, educational level, marital status, socio-economic level, and employment status were collected.

#### 2.2.2 Present Medical History

Data on complaints, onset, course, duration, precipitating factors, variation of symptoms, diurnal, weekly, weather and seasonal, medical care setting and specialities visited, previous investigations, management options, response, and body mass index were collected.

#### 2.2.3 Past Medical History

Data on medical comorbidities, drug history, allergies to food, non-food, or drug/chemical allergies, and surgical history were collected.

#### 2.2.4 Psychiatric History

Data on psychiatric comorbidities, history of substance abuse, and stressors were collected.

#### 2.2.5 Family History

Data on similar symptoms in family and psychiatric family history were collected.

#### 2.2.6 Personal Habits

Data on smoking and history of alcohol and substance misuse were collected.

### 2.3 The Patient Health Questionnaire

The somatic symptom severity of patients was recorded using the Arabic version of the Patient Health Questionnaire (PHQ-15) [16]. The PHQ-15 is one of the most suitable scales for assessing somatic symptoms in large-scale studies. It has well-established psychometric properties, measures relevant symptoms, and is relatively short. The Arabic version of the PHQ-15 has been validated and shown to be a reliable tool. It consists of a self-report questionnaire composed of 15 items, each scored between 0 to 2, resulting in a total possible severity score of 0 to 30. This was calculated by assigning scores of 0, 1, and 2, respectively, to the response categories of “not at all,” “bothered a little,” and “bothered a lot.” PHQ-15 scores of ≤ 4, 5, 10, and 15 represent the cut-off points for no/minimal, low, medium, and high somatic symptom severity, respectively.

### 2.4 The Arabic Version of the Toronto Alexithymia Scale

The Toronto Alexithymia Scale (TAS-20) [17] is a multidimensional self-report instrument consisting of 20 items, each of which is scored on a 5-point Likert scale, from 1 (strongly disagree) to 5 (strongly agree). The three measured variables include difficulty identifying feelings (DIF), difficulty describing feelings (DDF), and externally-oriented thinking (EOT) [17]. The total possible score is 20 to 100. DIF scores range from 7 to 35, DDF scores from 5 to 25, and EOT scores from 8 to 40. More serious alexithymia is indicated by higher scores. Respondents were classified into the following categories using the total TAS-20 score: alexithymic (scores> 61), borderline/intermediate (scores between 51 and 60), and non-alexithymic (scores < 50).

### 2.5 Statistical Analysis

Data were analyzed using IBM SPSS software package version 20.0. Qualitative data are presented as the number and percentage. The Kolmogorov-Smirnov test was used to validate the normality of distribution. Quantitative data are described using the range (minimum and maximum), mean, standard deviation, median, and interquartile range. Results were considered significant at the 5% level.

## 3 RESULTS

A total of 169 patients with MUPS recruited from tertiary care internal medicine and neuropsychiatry clinics were included in this study.

### 3.1 Socio-demographic Profile

The study included 176 female patients and 20 male patients (male-to-female ratio = 1:9). Participants were categorized into three age groups; young adults aged 18 to 35 years constituted 88.8% (n=174), middle-aged adults aged 36 to 45 years constituted 5.6% (n=11), and older adults aged 46 to 60 years constituted 5.6% (n=11). Concerning the place of origin of patients, 85.7% (n=168) were from a city, while 13.8% (n=27) were from the countryside and only 0.5% (n=1) were Bedouin. Detailed socio-demographic details of the enrolled patients are shown in Table **1**.

### 3.2 The Clinical Profile of Patients

Present somatic symptoms were assessed using the PHQ-15. The distributions of patients’ complaints are presented in Table **2**. “Feeling tired or having low energy” was the most common complaint observed, about which the patients were highly bothered. The second most common complaint was “Headaches,” followed by “Nausea, gas, or indigestion.”

The severity of somatic symptoms was measured using the PHQ-15 total score, according to which patients were distributed in the following four subgroups: minimal, low, medium, and high. In the enrolled subjects, 73.5% of the patients had a high somatic symptom severity, 17.9% had medium severity somatic symptoms, while 8% and 0.5% had low and minimal severity somatic symptoms, respectively (Table **3**).

Patients’ symptoms were further analysed for clinically pertinent parameters, including onset, course, diurnal variation, seasonality, and relation to the menstrual cycle in women, and precipitating factors. These details are presented in Table **4**.

The weight and height of the patients were recorded, and the body mass index was calculated (Table **5**). Patients were categorized into the following categories according to their scores: 36.2% of the patients had an ideal weight, while 30.6% and 28.1% were overweight and obese, respectively. The mean body mass index was 27.01 ± 6.09 (kg/m^2^).

Allergy history, including food allergy, non-food allergy, and sensitivity to chemicals/drugs, was recorded. Parallel to emerging MUPS, new sensitivities in the enrolled subjects were recorded in a significant portion. These results revealed that 5.6% of patients developed a new food allergy, 9.2% of patients developed a new category of allergies, and around 25% of patients developed one or more new sensitivities to chemicals and/or drugs (Table **6**).

Queries related to personal habits revealed that 4.1% of patients (n=8) were smokers, 1% (n=2) had a history of substance abuse, and 0.5% reported a history of alcohol intake.

Based on the total score, patients were divided into those who had alexithymia (49.5%), borderline/intermediate alexithymia (27.6%), and non-alexithymic (23%) (Table **7**).

The mean score was 59.24 ± 12.77, while the median was 60. For the TAS-20 subscales, patients had a mean score of 23.14 ± 6.87, 15.43 ± 4.60, and 20.67 ± 4.72 for the DIF, DDF, and EOT subscales, respectively (Table **8**).

Pearson’s correlation analysis revealed a weak positive correlation between the TAS-20 score and the severity of somatic symptoms as measured by the PHQ-15 (r=0.277, p<0.05). This positive correlation was interpreted as weak because Spearman’s correlation coefficient was above 0.2 but less than 0.4. Only the DIF dimension score of the TAS-20 showed a weak positive correlation with the PHQ-15 score (r=0.271, p<0.05); scores of the DDF and EOT dimensions were not significantly correlated with the PHQ-15 score (Table **9**).

A significant positive association was found between suicidal thoughts and/or death wishes and the total PHQ-15 score, highlighting the need for risk assessment in patients with MUPS. Patients who reported death wishes or suicidal thoughts had more severe somatic symptoms than those who did not (p<0.001), which was revealed by a linear regression analysis using death wishes and/or suicidal thoughts as the dependent variable and the PHQ-15 score as a covariate (Table **10**).

## 4 DISCUSSION

Mental health conditions are marked by an excessive emphasis on physical (somatic) symptoms that potentially cause anxiety and/or interfere with regular activities. Somatization, coupled with a severe cognitive disability, is one of the most common challenges in health care systems; hence their credible and accurate acquisition is critically appropriate. Somatoform signs are marked by medically unexplained signs, which tend to continue for a prolonged duration [18, 19]. A growing body of scientific data indicates that the term “functional” rather than“medically unexplained” is justified in these situations [20]. These diseases, which can have developmental, predisposing, and initiating causes, most likely have multifactorial causes. Of these, stress is the most prevalent, and these factors have also been related to toxic stress encountered in childhood [21, 22]. Sensitization of the central nervous system tends to be the central mechanism of functional disorders. This may induce a multitude of symptoms from the overactive autonomous nervous system, and this perspective has been supported by recent functional magnetic resonance imaging experiments [23]. Previous work has shown that symptoms in patients with functional disorders, particularly those that are associated with pain, can be largely attributed to central sensitization. In patients with MUPS, neuroimaging studies have shown associated changes in the gray matter of areas related to pain processing, including the periaqueductal gray matter, thalamus, somatosensory cortex, and insula. Increased levels of glutamate (the main excitatory amino acid) and decreased levels of gamma-aminobutyric acid (the main inhibitory amino acid) have been found in the insular cortex. Endogenous pain modulatory networks can be altered, leading to exaggerated pain on the application of a painful stimulus and even painful sensations from normally unpainful stimulation. The imbalance between neuronal networks that increase and decrease the nociceptive effect of such stimuli is considered a major component of the central sensitization phenomenon implicated in functional disorders [23, 24].

In primary care and student populations, somatization/somatic symptom reporting is often evaluated using the PHQ-15 [25]. The PHQ-15 is a self-administered scale that assesses somatic symptoms and has been regularly employed in medical studies and clinical practice [26]. This study aimed to determine the prevalence of alexithymia in patients with MUPS and to analyze the relationship between MUPS and socio-demographic variables.

The study recruited 196 patients with a broad socio-demographic profile, and no significant correlation was found between the socio-demographic attributes and the incidence of alexithymia in patients with MUPS.

Present somatic symptoms were assessed using the PHQ-15. The most common complaint for which the patients were highly bothered was “Feeling tired or having low energy,” followed by “headaches” and “nausea, gas, or indigestion.” In addition, 73.5% of patients had high severity somatic symptoms, 17.9% had medium severity somatic symptoms, and 8% and 0.5% had low and minimal somatic symptoms, respectively.

Most patients could not recall the precipitating factor responsible for the associated somatic symptoms and could not specify any specific time period during which the incidence of the stated symptoms reached peak intensity. The small group of enrolled patients who were able to specify the factors predominantly attributed the occurrence of somatic symptoms to stress. In addition, 89.3% of patients with MUPS had no chronic physical diseases, no food or non-food allergies, and no sensitivity to chemicals or specific drugs. Nearly half of the patients with MUPS had alexithymia, while nearly a quarter had borderline alexithymia or did not have alexithymia.

Emotional information is processed differently in patients with MUPS, which is relevant in the context of alexithymic deficiencies. For example, according to the Bioinformational Theory of Emotion, emotional memories are retained in perceptual circuits assembled of explanatory, meaning-related, and response-related information [27]. Stimulation of any of these elements can, to some extent, activate other parts of the circuit. In contrast to non-alexithymic people, those with alexithymia tend to have fewer cohesive memory networks for emotion. This could explain why patients with alexithymia have difficulty expressing their emotions accurately [28, 29].

Recent research studying alexithymia from a non-emotional perspective has suggested that it could instead be related to difficulty interpreting signals coming from various body organs. This novel area of research has replaced the traditional view of alexithymia as a defective capacity to process emotions, yet further research is still required [30].

The present study found a high prevalence of alexithymia (49.5%) and identified culture-sensitive socio-demographic characteristics in patients with MUPS. Observational studies across different cultures and ethnicities have yielded variable results. This diversity further complicates the complex psychological construct of alexithymia and emphasizes the influence of culture on the mental representation of psychological suffering associated with difficulty in processing, understanding, and expressing emotions [31-34].

Notably, in alexithymia, a “decoupling” between subjective emotional perception and mechanisms of emotional appeals, such as autonomic somatic symptoms and facial expression, has been reported by several studies, which could explain the observed effect in the studied group of patients [35]. This decoupling may be a defining characteristic of alexithymia and could be responsible for the propensity of these individuals to misattribute disease, rather than emotion, to interoceptive stimuli [36]. This notion has been supported by emerging neuroimaging evidence that has shown inadequate connectivity between brain regions involved in the development of various emotional aspects in these patients [37-39].

Although previous work has emphasized the role of symptoms involved in classifying somatic symptoms to better explain what patients present to physicians, the community could also play an important role in increasing the general understanding of somatic symptoms. Population-based experiments involving single communities could offer more support for the consideration of normative symptoms in non-clinical populations and help identify somatization symptoms [40]. One strength of the present study is that it highlights the high prevalence of alexithymia in patients with MUPS in a specific cultural and ethnic background that may be different from Western cultures, in which the expression of emotions and ability to show emotions is different.

This challenge may be an intrinsic deficit or may be indicative of the ability to control strong and unwanted feelings. In the long-term, the avoidance of recognizing, explaining, and analyzing emotions can hinder the regulation of emotional control, suppress sensitivity and annihilation of a negative impact, and increase distrust in inner interactions. Alexithymia is challenging for psychotherapy. Moreover, recent works have been revealed a positive effect of long-term dynamic psychotherapy [41] and short-term dynamic therapy [42, 43]. Alexithymia has an effect on psychotherapeutic outcomes [44, 45]. These findings highlight the importance of raising awareness about alexithymia in public health settings.

## LIMITATIONS

Our study is limited by the small sample size, which meant that statistical significance could not be tested; however, the prevalence of alexithymia in a representative sample of the population was provided. Furthermore, the socio-demographic biases associated with the psychometric assessment of patients with alexithymia could not be removed due to the consecutive subcategorization of groups and weak statistical results. The inclusion of psychometric tools and a questionnaire to evaluate emotional regulations, disease conceptualization, and somatization as a defence mechanism provides a platform for future studies and deepens our understanding of alexithymia, which is an old term yet still enigmatic and scientifically vague. We did not include psychological testing for diverse personality traits and constructs; future studies could add this depth to the analysis by bridging the gap between alexithymia and MUPS. Although the present study found a significant association between the severity of somatic symptoms in patients with MUPS and death wishes, we did not use psychometric scales to further assess the risk of suicide.

## CONCLUSION

It is important to perform in-depth investigations on the causal mechanisms underlying alexithymic emotion regulation deficits in a large group of patients. People with alexithymia may not be able to adequately perceive and interpret emotions but may have adapted to suppress them, probably because they have insufficient emotion control. Strong and painful feelings may seem daunting to them, and there arises the possibility that patients with alexithymia have learned to suppress their feelings to the point that they do not recognize them or differentiate them from other bodily stimuli. Our findings indicate that therapeutic strategies that encourage emotional control, emotional resilience, therapeutic flexibility, and a sense of effectiveness in coping with interactions could help to overcome alexithymia symptoms and their effect on mental health, which in turn could reduce MUPS.

## AUTHORS' CONTRIBUTIONS

All authors equally contributed to conceptualization, study design, data analysis, arrangement of tables, analysis, interpretation of data, and writing and reviewing the manuscript.

## Figures and Tables

**Table 1:** Distribution of the studied cases according to socio-demographic data (N = 196).

**Sociodemographic Characteristics**	**No.**	**%**
**Gender**		
Male	20	10.2
Female	176	89.8
**Age (Years)**		
18 – 35	174	88.8
36 – 45	11	5.6
46 – 60	11	5.6
**Marital Status**		
Single	106	54.1
Married	80	40.8
Divorced	8	4.1
Widowed	2	1.0
**Hometown/Origin**		
City	168	85.7
Countryside	27	13.8
Bedouin	1	0.5
**Habitat**		
Same as hometown	123	62.8
Moved	73	37.2
**Living Situation**		
Alone	11	5.6
With Family	181	92.3
With Extended Family/Relatives	4	2.0
**Education Level**		
Secondary School	2	1.0
Diploma	4	2.0
University	190	96.9
**Employment Status**		
Unemployed	70	35.7
Casual employee	21	10.7
Permanent or fixed-term employee	101	51.5
Early Retirement (<60 years old)	4	2.0
**Socioeconomic Level**		
Low	6	3.1
Low-Middle	97	49.5
Middle	85	43.4
High	8	4.1

**Table 2:** Patients complaints according to items of PHQ-15 (n = 196).

**PHQ-15 Items**	**Not** **Bothered**		**Bothered** **A little**		**Bothered** **A lot**	
	**No.**	**%**	**No.**	**%**	**No.**	**%**
Feeling tired or having low energy	7	3.6	19	9.7	170	86.7
Headaches	15	7.7	87	44.4	94	48.0
Nausea, gas, or indigestion	18	9.2	58	29.6	120	61.2
Trouble sleeping	24	12.2	63	32.1	109	55.6
Pain in arms, legs or joints	26	13.3	64	32.7	106	54.1
Back pain	27	13.8	73	37.2	96	49.0
Stomach pain	33	16.8	81	41.3	82	41.8
Feeling your heart pound or race	38	19.4	75	38.3	83	42.3
Constipation, loose bowels, or diarrhea	38	19.4	73	37.2	85	43.4
Dizziness	40	20.4	86	43.8	70	35.7
Menstrual cramps or other problems with periods	58	29.6	70	35.7	68	34.7
Chest pain	65	33.2	81	41.3	50	25.5
Shortness of breath	69	35.2	59	30.1	68	34.7
Sexual intercourse pain or problems	144	73.5	33	16.8	19	9.7
Fainting spells	167	85.2	27	13.8	2	1.0

**Table 3:** Distribution of the studied cases according to somatic symptoms severity and their perceived difficulty (n = 196).

-	**No.**	**%**
**Total PHQ-15 Score**		
Minimal (0 – 4)	1	0.5
Low (5 – 9)	16	8.2
Medium (10 – 14)	35	17.9
High (15 – 30)	144	73.5
Min. – Max.	3 – 28	
Mean ± SD.	17.31 ± 5.02	
Median	18	
**How difficult have these problems made it for you to do your work, take care of things at home, or get along with other people?**		
Not Difficult at all	3	1.5
Somewhat Difficult	97	49.5
Very Difficult	65	33.2
Extremely Difficult	31	15.8

**Table 4:** Symptoms analysis of the studied cases (n = 196).

**Symptoms Analysis**	**No.**	**%**
**Onset**		
Acute	33	16.8
Gradual	163	83.2
**Course**		
Progressive	55	28.1
Intermittent	111	56.6
Regressive	3	1.5
Stationary	27	13.8
**Symptoms Variation**		
**Diurnal Variation**		
No variation	98	50.0
Morning	34	17.4
Afternoon	21	10.7
Evening	43	21.9
**Weekly Variation**		
No variation	145	74.0
Weekdays	42	21.4
Weekends	9	4.6
**Weather/Seasonal Variation**		
No variation	135	68.9
Hot Weather/Summer	34	17.3
Cold Weather/Winter	27	13.8
**Menstrual Variation (n=176)**		
No variation	63	35.8
Premenstrual	70	39.8
During Mensis	35	19.9
After end of Mensis	8	4.5
**Precipitating Factors**		
No	101	51.5
Yes	95	48.5
**With Precipitating Factors (n=95)**		
Personal Stressors (Unspecified)	36	18.4
Work and Financial Stressors	16	8.2
Relational or Marital Problems	14	7.1
Pregnancy and Delivery	11	5.6
Educational Stressors	9	4.6
Death or illness of a family member	4	2.0
Others	5	5.0
**Duration (months)**		
Min. – Max.	3 – 22	
Mean ± SD.	33.90 ± 29.88	
Median	18	

**Table 5:** Distribution of the studied cases according to BMI (n = 196).

**Body Mass Index (BMI)**	**No.**	**%**
**BMI (kg/m^2^)**		
Underweight (<18.5)	10	5.1
Ideal (18.5 – 24.9)	71	36.2
Overweight (25.0 – 29.9)	60	30.6
Obese (≥30)	55	28.1
Min. – Max.	14.50 – 46.10	
Mean ± SD.	27.01 ± 6.09	
Median	26.05	

**Table 6:** Allergy/Sensitivities status of the sample (n = 196).

**Allergy/Sensitivities**	**Since Birth**		**New**		**No Sensitivity**	
	**No.**	**%**	**No.**	**%**	**No.**	**%**
Food Allergy	12	6.1	11	5.6	173	88.3
Non-food Allergy	63	32.1	18	9.2	115	58.7
Sensitivity to Chemicals/Drugs	21	10.7	48	24.5	127	64.8

**Table 7:** Distribution of the studied cases according to TAS-20 score (n = 196).

**TAS-20 score**	**No.**	**%**
No Alexithymia (≤50)	45	22.9
Borderline or intermediate alexithymia (51 –60)	54	27.6
Alexithymia (≥61)	97	49.5

**Table 8:** TAS-20 subscales scores in patients with MUPS (N = 196).

-	Mean ± SD.	Median
Difficulty identifying feelings (DIF)	23.14 ± 6.87	23
Difficulty describing feelings (DDF)	15.43 ± 4.60	15
Externally oriented thinking (EOT)	20.67 ± 4.72	21
Total Toronto Alexithymia Scale (TAS-20)	59.24 ± 12.77	60

**Table 9:** Correlation between PHQ-15, TAS-20 and individual dimensions scores of Alexithymia (DIF,DDF and EOT) in patients with MUPS (N = 196).

-	Patient Health Questionnaire (PHQ-15)
	r_s_
Difficulty identifying feelings (DIF)	0.271* 0.182 0.177 0.277*
Difficulty describing feelings (DDF)	
Externally oriented thinking (EOT)	
Total Toronto Alexithymia Scale (TAS-20)	

r_s_: Spearman Correlation Coefficient (weak correlation r_s_ 0.2 to 0.39 ; strong correlation r_s_ 0.4 to 0.69)

*p<0.05

**Table 10:** Association between having death wishes and/or suicidal thoughts and PHQ-15 (n=196).

Patients who had Death Wishes and/or Suicidal Thoughts	N	PHQ-15 Score	Test of Sig.	P
		Mean ± SD.		
No	88	15.91 ± 5.32	t=3.637*	<0.001*
Yes	108	18.45 ± 4.48		

t: Student t-test

p: p value for association between the studied categories

*: Statistically significant at p < 0.05

## Data Availability

The datasets used in the current study can be provided by the corresponding author upon reasonable request.

## References

[1] Kellner R. (1990). Somatization. Theories and research.. J. Nerv. Ment. Dis..

[2] Baitha U., Deb K.S., Ranjan P., Mukherjee A., Bauddh N.K., Kaloiya G.S., Kumar A., Jadon R.S. (2019). Estimated prevalence of medically unexplained physical symptoms in the medicine outpatient department of a tertiary care hospital in India.. Gen. Hosp. Psychiatry.

[3] Evangelidou S., NeMoyer A., Cruz-Gonzalez M., O’Malley I., Alegría M. (2020). Racial/ethnic differences in general physical symptoms and medically unexplained physical symptoms: Investigating the role of education.. Cultur. Divers. Ethnic Minor. Psychol..

[4] Walker E.A., Katon W.J., Neraas K., Jemelka R.P., Massoth D. (1992). Dissociation in women with chronic pelvic pain.. Am. J. Psychiatry.

[5] Nixon A.E., Mazzola J.J., Bauer J., Krueger J.R., Spector P.E. (2011). Can work make you sick? A meta-analysis of the relationships between job stressors and physical symptoms.. Work Stress.

[6] van Dijk S.D.M., Hanssen D., Naarding P., Lucassen P., Comijs H., Oude Voshaar R. (2016). Big Five personality traits and medically unexplained symptoms in later life.. Eur. Psychiatry.

[7] Kellner R., Abbott P., Winslow W.W., Pathak D. (1987). Fears, beliefs, and attitudes in DSM-III hypochondriasis.. J. Nerv. Ment. Dis..

[8] Manu P., Manu P. (1998). Functional somatic syndromes: etiology, diagnosis and treatment..

[9] Kooiman C.G., Bolk J.H., Rooijmans H.G., Trijsburg R.W. (2004). Alexithymia does not predict the persistence of medically unexplained physical symptoms.. Psychosom. Med..

[10] Taylor G.J., Bagby R.M., Parker J.D. (1999). Disorders of affect regulation: Alexithymia in medical and psychiatric illness..

[11] Hadji-Michael M., McAllister E., Reilly C., Heyman I., Bennett S. (2019). Alexithymia in children with medically unexplained symptoms: a systematic review.. J. Psychosom. Res..

[12] Kooiman C.G. (1998). The status of alexithymia as a risk factor in medically unexplained physical symptoms.. Compr. Psychiatry.

[13] Carrozzino D., Porcelli P. (2018). Alexithymia in gastroenterology and hepatology: a systematic review.. Front. Psychol..

[14] Khosravani V., Samimi Ardestani S.M., Alvani A., Amirinezhad A. (2020). Alexithymia, empathy, negative affect and physical symptoms in patients with asthma.. Clin. Psychol. Psychother..

[15] Aival-Naveh E., Rothschild‐Yakar L., Kurman J. (2019). Keeping culture in mind: A systematic review and initial conceptualization of mentalizing from a cross-cultural perspective.. Clin. Psychol. Sci. Pract..

[16] AlHadi A.N., AlAteeq D.A., Al-Sharif E., Bawazeer H.M., Alanazi H., AlShomrani A.T., Shuqdar R.M., AlOwaybil R. (2017). An arabic translation, reliability, and validation of Patient Health Questionnaire in a Saudi sample.. Ann. Gen. Psychiatry.

[17] El Abiddine F.Z., Dave H., Aldhafri S., El-Astal S., Hemaid F., Parker J.D. (2017). Cross-validation of the 20-item Toronto Alexithymia Scale: Results from an Arabic multicenter study.. Pers. Individ. Dif..

[18] Körber S., Frieser D., Steinbrecher N., Hiller W. (2011). Classification characteristics of the Patient Health Questionnaire-15 for screening somatoform disorders in a primary care setting.. J. Psychosom. Res..

[19] Hiller W., Fichter M.M. (2004). High utilizers of medical care: a crucial subgroup among somatizing patients.. J. Psychosom. Res..

[20] Bègue I., Adams C., Stone J., Perez D.L. (2019). Structural alterations in functional neurological disorder and related conditions: a software and hardware problem?. Neuroimage Clin..

[21] Lykkegaard J., Rosendal M., Brask K., Brandt L., Prior A. (2018). Prevalence of persons contacting general practice for psychological stress in Denmark.. Scand. J. Prim. Health Care.

[22] Winding T.N., Andersen J.H. (2019). Do negative childhood conditions increase the risk of somatic symptoms in adolescence? - a prospective cohort study.. BMC Public Health.

[23] Harte S.E., Harris R.E., Clauw D.J. (2018). The neurobiology of central sensitization.. J. Appl. Biobehav. Res..

[24] Donnelly C.R., Andriessen A.S., Chen G., Wang K., Jiang C., Maixner W., Ji R.R. (2020). Central nervous system targets: glial cell mechanisms in chronic pain.. Neurotherapeutics.

[25] Jasper F., Hiller W., Rist F., Bailer J., Witthöft M. (2012). Somatic symptom reporting has a dimensional latent structure: results from taxometric analyses.. J. Abnorm. Psychol..

[26] Kroenke K., Spitzer R.L., Williams J.B. (2002). The PHQ-15: validity of a new measure for evaluating the severity of somatic symptoms.. Psychosom. Med..

[27] Lang P.J. (1979). Presidential address, 1978. A bio-informational theory of emotional imagery.. Psychophysiology.

[28] Connelly M., Denney D.R. (2007). Regulation of emotions during experimental stress in alexithymia.. J. Psychosom. Res..

[29] Eastabrook J.M., Lanteigne D.M., Hollenstein T. (2013). Decoupling between physiological, self-reported, and expressed emotional responses in alexithymia.. Pers. Individ. Dif..

[30] Brewer R., Cook R., Bird G. (2016). Alexithymia: a general deficit of interoception.. R. Soc. Open Sci..

[31] Ryder A.G., Sunohara M., Dere J., Chentsova-Dutton Y.E. (2018). The cultural shaping of alexithymia. Alexithymia..

[32] Kamm J.S. High alexithymia.. https://d-nb.info/1147255458/34.

[33] Dreher A., Hahn E., Diefenbacher A., Nguyen M.H., Böge K., Burian H., Dettling M., Burian R., Ta T.M.T. (2017). Cultural differences in symptom representation for depression and somatization measured by the PHQ between Vietnamese and German psychiatric outpatients.. J. Psychosom. Res..

[34] Kamm S.G., Sepulveda E., Brosig B. (2016). Adolescent alexithymia research: Indigenous sample compared to Hispanic sample in Southern Chile.. Psychology (Irvine).

[35] Roedema T.M., Simons R.F. (1999). Emotion-processing deficit in alexithymia.. Psychophysiology.

[36] Byrne N., Ditto B. (2005). Alexithymia, cardiovascular reactivity, and symptom reporting during blood donation.. Psychosom. Med..

[37] Chalah M.A., Kauv P., Palm U., Lefaucheur J.P., Hodel J., Créange A., Ayache S.S. (2020). Deciphering the neural underpinnings of alexithymia in multiple sclerosis.. Neurosci. Lett..

[38] Patrikelis P., Lucci G., Alexoudi A., Korfias S., Messinis L., Nasios G., Papasilekas T., Sakas D., Gatzonis S. (2019). Addressing evidence linking secondary alexithymia to aberrant humor processing.. Behav. Neurol..

[39] Terock J., Frenzel S., Wittfeld K., Klinger-König J., Janowitz D., Bülow R., Hosten N., Völzke H., Grabe H.J. (2020). Alexithymia Is Associated with Altered Cortical Thickness Networks in the General Population.. Neuropsychobiology.

[40] Mostafaei S., Kabir K., Kazemnejad A., Feizi A., Mansourian M., Hassanzadeh Keshteli A., Afshar H., Arzaghi S.M., Rasekhi Dehkordi S., Adibi P., Ghadirian F. (2019). Explanation of somatic symptoms by mental health and personality traits: application of Bayesian regularized quantile regression in a large population study.. BMC Psychiatry.

[41] Khademi M., Hajiahmadi M., Faramarzi M. (2019). The role of long-term psychodynamic psychotherapy in improving attachment patterns, defense styles, and alexithymia in patients with depressive/anxiety disorders.. Trends Psychiatry Psychother..

[42] Zarei E., Dortaj F. (2021). Effectiveness of intensive short-term dynamic psychotherapy on Alexithymia in Rheumatoid Arthritis patients.. J Res Behavio Sci.

[43] Bressi C., Fronza S., Minacapelli E., Nocito E.P., Dipasquale E., Magri L., Lionetti F., Barone L. (2017). Short-Term Psychodynamic Psychotherapy with Mentalization-Based Techniques in Major Depressive Disorder patients: Relationship among alexithymia, reflective functioning, and outcome variables - A Pilot study.. Psychol. Psychother..

[44] Probst T., Sattel H., Gündel H., Henningsen P., Kruse J., Schneider G., Lahmann C. (2017). Moderating effects of alexithymia on associations between the therapeutic alliance and the outcome of brief psychodynamic-interpersonal psychotherapy for multisomatoform disorder.. Front. Psychiatry.

[45] Blaettler L.T., Stewart J.A., Gubler D.A., Egloff N., von Känel R., Grosse Holtforth M. (2019). Alexithymia moderates effects of psychotherapeutic treatment expectations on depression outcome in interdisciplinary chronic pain treatment.. J. Psychosom. Res..

